# Validation of the French Version of the Child Posttraumatic Stress Checklist in French School-Aged Children

**DOI:** 10.3389/fpsyt.2021.678916

**Published:** 2021-08-20

**Authors:** Morgane Gindt, Aurelien Richez, Michèle Battista, Roxane Fabre, Susanne Thümmler, Arnaud Fernandez, Florence Askenazy

**Affiliations:** ^1^Service Universitaire de Psychiatrie de l'Enfant et de l'Adolescent, Hôpitaux Pédiatriques de Nice CHU-Lenval, Nice, France; ^2^Université Côte d'Azur, CoBTek, FRIS, Nice, France; ^3^Centre Expert du Psychotrauma PACA Corse, Nice, France; ^4^Département de Sante Publique, Centre Hospitalier Universitaire de Nice, Université Cote d'Azur, Nice, France

**Keywords:** CPC, french, validation, school-aged children, PTSD, psychiatry, psychometric properties

## Abstract

**Background:** The child posttraumatic stress disorder checklist (CPC) updated to DSM-5 is a questionnaire aimed to assess posttraumatic stress disorder (PTSD) symptoms in children. It is available in both parents and child versions. The back-translation method has been used for the French translation of the CPC. It has not been yet validated in French-speaking populations. The aim of this study was to assess the psychometric properties and the validity of the CPC in a sample of French-speaking schoolchildren and their parents.

**Methods:** The sample was composed by 176 children outpatients implicated in the Nice terrorist attack (14 July 2016) aged 7–17 (mean = 11.68 years, SD = 2.63 months) and 122 parents. Cronbach's alpha was used to test CPC internal consistency. The Spearman-correlation coefficient was performed between the French version of the CPC and the Kiddie Schedule for Affective Disorders and Schizophrenia Present and Lifetime version (K-SADS-PL) to assess the convergent validity. An ROC curve was constructed to verify the validity of the cutoff scores. An evaluation of the sensitivity and specificity of each score and a comparison with the diagnosis of the K-SADS-PL were made. Finally, a principal component analysis with varimax rotation was computed to analyze the structure of the French version of the CPC.

**Results:** Cronbach's alpha coefficient was 0.90 for child version and 0.91 for parent version of the CPC. There was a statistical correlation between the K-SADS-PL for PTSD and the total score of CPC for the child version (*r* = 0.62; *p* < 0.001) and for the parent version (*r* = 0.55; *p* < 0.001). The sensitivity and specificity of the children version with a threshold of >20 were 73.1 and 84.7%, respectively, using the K-SADS-PL as the diagnostic reference for PTSD. Concerning the parent version, using the same recommended cutoff score, the sensitivity, and specificity were 77 and 80.5%, respectively.

**Conclusions:** The psychometric properties of the French CPC are good. This questionnaire appears to be valid and should be used in French-speaking children.

## Introduction

Since the consideration of the specific problem of posttraumatic stress disorder (PTSD) in children in the DSM-III-R (1), the vision of the consequences of a psychotrauma in children has changed. Many studies have shown that traumatic experience in childhood affects the overall development of the child (social development, emotional development, or cognitive development) (2, 3).

In DSM-5 (4), specific clusters were defined according to the age of the child, in particular for young children. The American Academy of Child and Adolescent Psychiatry (5, 6) recommends emphasizing the use of different sources of information (children, parents, and/or caregiver) in order to establish the diagnosis of this disorder. Indeed, children may not reveal their traumatic experience to their parents (7) or parents may minimize it.

Pediatric PTSD includes four main categories of symptoms: revival of the event; avoidance behaviors; impaired cognition and mood; and neuro-vegetative overactivation. These are the same groups of symptoms as those seen in adults. However, their clinical expressions tend to be different: traumatic games, return of developmental fears previously extinguished, or even bedwetting and encopresis (8, 9).

An early diagnosis of PTSD in children allows the practitioner to offer specific and rapid treatment, in order to avoid the chronicity of this disorder and the associated generalization mechanisms. If the symptoms of PTSD are not rapidly treated, developmental damages may appear: self-esteem disorder, personality traits, or cognitive impairments.

In order to help diagnose PTSD, in addition to clinical interviews, several tools can be used. To assess the presence and intensity of posttraumatic stress symptoms, semi-structured interviews or self- or hetero-questionnaires can be offered. These different techniques have some advantages and disadvantages (10). Self-administered questionnaires are quick and easy to administer. Conversely, semi-structured interviews are relatively long and require training. Nevertheless, they allow a more detailed assessment of symptoms and give better information (10).

Scheeringa (11), in agreement with the DSM-5, developed the child PTSD checklist (CPC). This questionnaire assesses the symptoms of PTSD, according to the DSM-5, as well as their frequency in children from 7 to 18 years, following a traumatic event. The lowest age of 7 years has been carried over from the original validation of the English version of the scale (11). Before this age, taking a self-administered questionnaire individually turns out to be impossible because of the lack of reading skills.

It has a child version and a parent version. These versions are built on the same model: a first section evaluating the presence of traumatic events in the child's life (direct or indirect exposure); if an event is checked, the following two sections are then proposed. A second section assesses the frequency of the symptoms (21 questions) and the last one the functional impairment (6 questions). Children and parents are asked to answer each question using a Likert scale of 0 (Never) to 4 (Daily). The cutoffs are 20 for the intensity of the symptoms and 4 for the functional discomfort. The completion time is 15–20 min. It is generally recommended to pass it with a clinician (psychologist or child and adolescent psychiatrist).

Currently, the only specific scale validated to assess the pediatric PTSD in French-speaking children is the Child Post-Traumatic Stress Reaction Index (CPTS-RI) (12). This questionnaire was validated in 2014 by Olliac et al. It helps to highlight the presence of PTSD symptoms and to indicate the intensity of these symptoms. However, this questionnaire is based on the DSM-IV and therefore does not take into account the changes brought about by the DSM-5.

The aim of this article is to validate and examine the psychometric properties of the CPC French version, using the data collected in the “14-7” Program, conducted with children exposed to the Nice (France) terrorist attack, in 2016 (13).

## Methods

### Participants and Procedures

The data used for the validation of the French CPC were obtained from a study carried out in the aftermath of the terrorist attack of July 14, 2016, in Nice, France, which resulted in 86 deaths and ~30,000 people exposed to the attack. A total of 176 children aged 7–17 (mean = 11.68 years, SD = 2.63 months) were recruited (CPC child version). All of them were exposed to a DSM-5 type 1 traumatic event. Among them, 86 were girls (49%). A total of 122 parents were also included to evaluate the psychometric properties of the CPC parent/caregiver version.

The French Consultative Committee for the Protection of Individuals in Biomedical Research (national ethics committee) approved all procedures of the present study (number 2017-A02212-51). An informed consent was signed by the parents and the child.

### Measures

The team of the pediatric psychotrauma center of Nice (France), using the Back Translation Method, carried out the French translation (14).

The K-SADS-PL (Kiddie—Schedule for Affective Disorders and Schizophrenia, Present, and Lifetime version) is a semi-structured diagnostic interview for children aged 6–18 (15), in agreement with the DSM-5. It is carried out by questioning the parent(s) and child, in order to integrate them into a summary note, which includes the report of the parent(s), the child's report, and the clinical observations during the interview. The interview covers both current issues (including why the family is seeking an assessment), as well as the latest episodes of the disorder. Most articles use a rating scale with three levels of severity (not present, subliminal, and threshold, which combines both moderate and severe presentations).

The use of the K-SADS-PL makes it possible to take into account the absence of redundancy between the questions due to an oral evaluation vs. written evaluation and the comparison between the oral responses of children and parents to their specific versions of the CPC. In addition, the K-SADS-PL is one of the few clinical instruments available in the French language evaluating pediatric PTSD according to DSM-5.

### Statistical Analyses

Principal component analysis was first carried out. The numbers of dimensions selected were evaluated looking at the eigenvalue diagram. A factorial analysis with an orthogonal rotation (Varimax) was performed (16). The rates of variance explained by the dimensions selected were determined. The loading values were checked in the case of each dimension. Only items that were substantially loaded (>|0.40|) on a single factor were selected.

Each dimension that emerged from the principal component analysis was used to define a subscale. The score obtained on each subscale was computed by summing up the answers to the items comprising the subscale. Items were scored from 0 to 4. The floor and ceiling effects were evaluated.

Internal consistency was tested by Cronbach's alpha. A coefficient higher than 0.60 was considered as good (16).

The Spearman-correlation coefficient evaluated concurrent validity between the K-SADS-PL for PTSD and the CPC score.

To prove the validity of the cutoff scores, an ROC curve was constructed which evaluated the sensitivity and specificity of each score compared to the diagnosis with the K-SADS-PL. A total severity cutoff score of three points with the K-SADS-PL was chosen, as it corresponds to the cutoff for clinical diagnosis. Then, we analyzed the ROC curve with the CPC cutoff >20 as recommended by Scheeringa (11).

To determine the link between the different scores and sex and gender in child version, Pearson correlation and Student *t*-test were used.

All analyses were performed using child version in the first time and parent version in the second time.

All statistical analyses were conducted using R software version 3.6.1 (The R Foundation for Statistical Computing, Vienna, Austria) with a statistical threshold for significance set to 0.05 (two-tailed).

## Results

### Factor Validity

For child version, PCA of the 21 items explained 43% of the variance with four factors (Table 1). The score obtained by summing up the 21 items ranged from 0 to 84, and the mean score was 23.6 (SD = 16.5). No floor or ceiling effects beyond the 15% threshold were observed.

**Table 1:** PCA for children and parents. Matrix of items.

	**Children**		**Parents**			
	**Factor 1**	**Factor 2**	**Factor1**	**Factor 2**	**Factor 3**	
Item 1	0.720	0.116	0.579	0.105	0.196	Repetitive memories
Item 2	0.679	0.145	0.553	0.358	0.229	Nightmares
Item 3	0.587	0.111	0.480	0.329	0.201	Derealization
Item 4	0.591	0.106	0.402	0.415	0.315	Freezing
Item 5	0.756	–	0.667	0.368	–	Emotional trouble
Item 6	0.727	−0.105	0.536	0.485	0.278	Physical disturbance
Item 7	0.802	0.177	0.354	0.596	0.333	Negative emotions
Item 8	0.555	–	0.394	0.373	0.242	Avoidance of conversations
Item 9	0.523	–	0.692	0.170	0.137	Avoidance of places or objects
Item 10	–	0.167	0.208	0.115	–	Difficulty remembering
Item 11	0.559	0.191	0.340	0.506	0.154	Negative beliefs
Item 12	0.447	0.105	0.150	0.430	0.232	False thoughts
Item 13	0.553	–	0.543	0.123	–	Anhedonia
Item 14	0.233	–	–	0.243	0.532	Distance from relatives
Item 15	0.493	0.174	0.222	0.118	0.965	Positive emotional difficulties
Item 16	0.648	0.115	0.289	0.509	0.441	Irritability
Item 17	0.309	0.949	–	0.646	–	Imprudence
Item 18	0.489	–	0.850	–	0.169	State of emergency
Item 19	0.621	–	0.685	0.302	0.177	Startle reaction
Item 20	0.571	0.241	0.297	0.387	0.418	Concentration difficulties
Item 21	0.615	–	0.451	0.364	0.400	Sleep disturbances
Eigenvalue	7.78	1.28	8.47	1.69	1.23	
% of variance	37.1	6.1	40.3	8.1	5.9	

The first factor consisted of the four DSM-5 symptoms: revival of the event; avoidance behaviors; impaired cognition and mood; and neuro-vegetative overactivation (items 1, 2, 3, 4, 5, 6, 7, 8, 9, 11, 12, 13, 15, 16, 18, 19, 20, and 21). It explained 37% of the variance. The score obtained by summing up the 18 items ranged from 0 to 72, and the mean score was 21.4 (SD = 15.6). No floor or ceiling effects were observed.

The second factor was represented only by item 17 which explained 6% of the variance. The mean score was 0.62 (SD = 1.12) and ranged from 0 to 4. One hundred twenty children (68.6%) responded the lower response.

Items 10 and 14 were not included in a factor because the factor loading was low on the two dimensions that emerged.

For parent/caregiver version, PCA of the 21 items explained 54% of the variance with three factors (Table 1). The score obtained by summing up the 21 items ranged from 0 to 84, and the mean score was 23.2 (SD = 16.6). No floor or ceiling effects were observed (Table 1).

The first factor explained 40% of the variance and consists of items 1, 2, 3, 5, 6, 8, 9, 13, 18, 19, and 21. This factor reflected symptoms of reexperiencing, avoidance, emotional and physical disturbance, anhedonia, nightmares, and sleep disturbances. The score obtained by summing up the 11 items ranged from 0 to 44, and the mean score was 14.0 (SD = 10.3). No floor or ceiling effects were observed.

The second factor explained 8% of the variance with items 4, 7, 11, 12, 16, and 17. They corresponded to symptoms of irritability and negative emotional state, negative beliefs and false thoughts, and freezing. The score obtained by summing up the six items ranged from 0 to 24, and the mean score was 5.7 (SD = 4.0). No floor or ceiling effects were observed.

The third factor consisted of items 14, 15, and 20, explained 6% of the variance, and was related to the distance from relatives (family and friends), attentional difficulties, and positive emotional difficulties. The score obtained by summing up the three items ranged from 0 to 12, and the mean score was 2.9 (SD = 2.8). Thirty-three parents (27.3%) had the lower score possible.

Item 10 was not included in a factor because the factor loading was low on the three dimensions that emerged.

### Internal Consistency

Cronbach's alpha for the total CPC was high and homogeneous for the child version (0.90). The first factor had also a good internal consistency (Cronbach's alpha = 0.91). For parents' version, the Cronbach's alphas were 0.92 for the total scale, 0.90 for the first factor, 0.80 for the second factor, and 0.67 for the third factor.

### Concurrent Validity

A positive correlation between the K-SADS-PL for PTSD and the total score of CPC was found for the child version (*r* = 62; *p* < 0.001) and for the parent/caregiver version (*r* = 0.55; *p* < 0.001).

For child version, the score total and the first factor were not associated with age and gender. The second factor was not associated with age, but was associated with gender with a lower score for girls (mean = 0.8, SD = 1.3 vs. mean = 0.4, SD = 0.9, *p* = 0.010).

### Receiver Operating Characteristic (ROC) Curve

**Figure 1:**
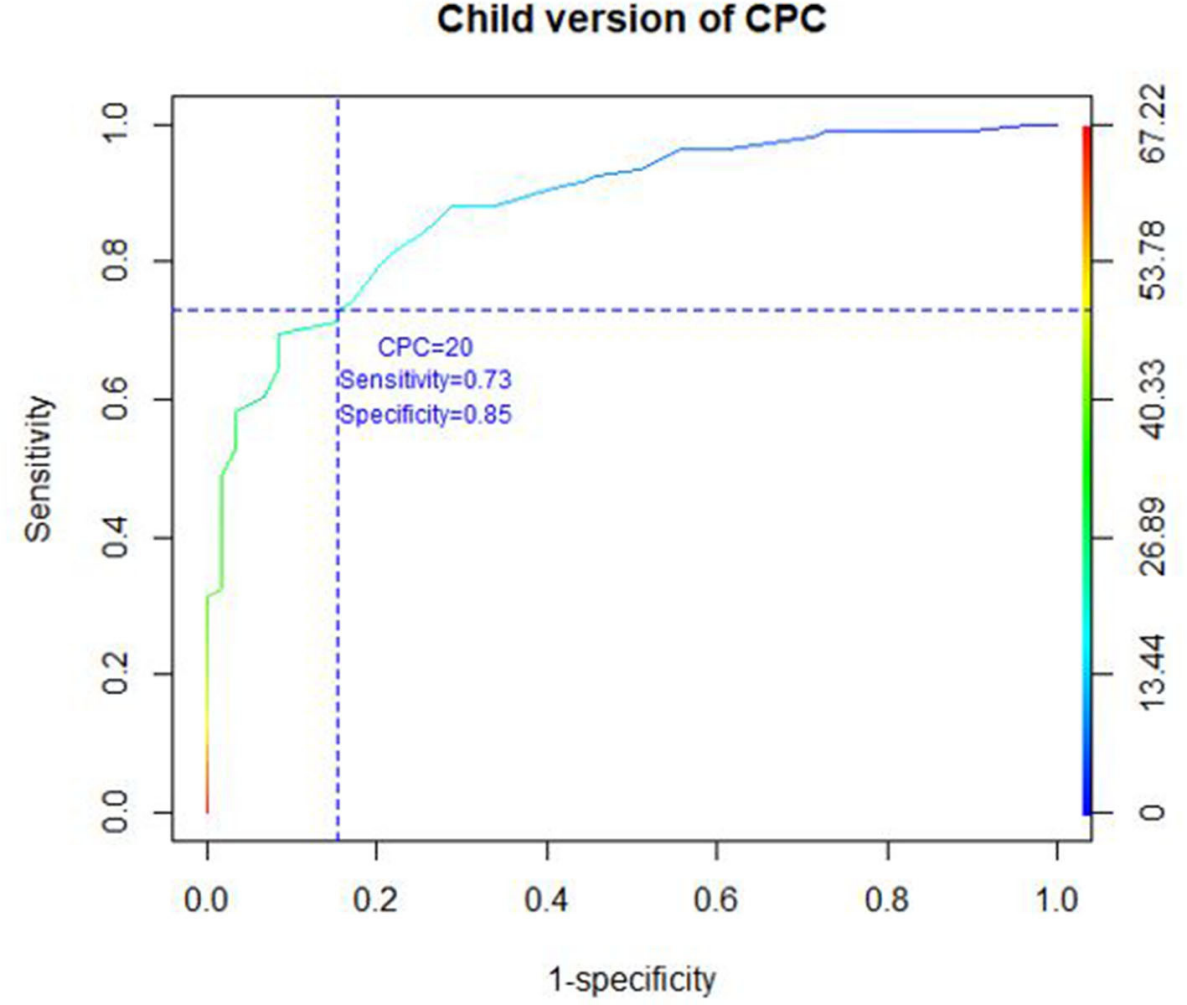
ROC CURVE child version of CPC.

**Figure 2:**
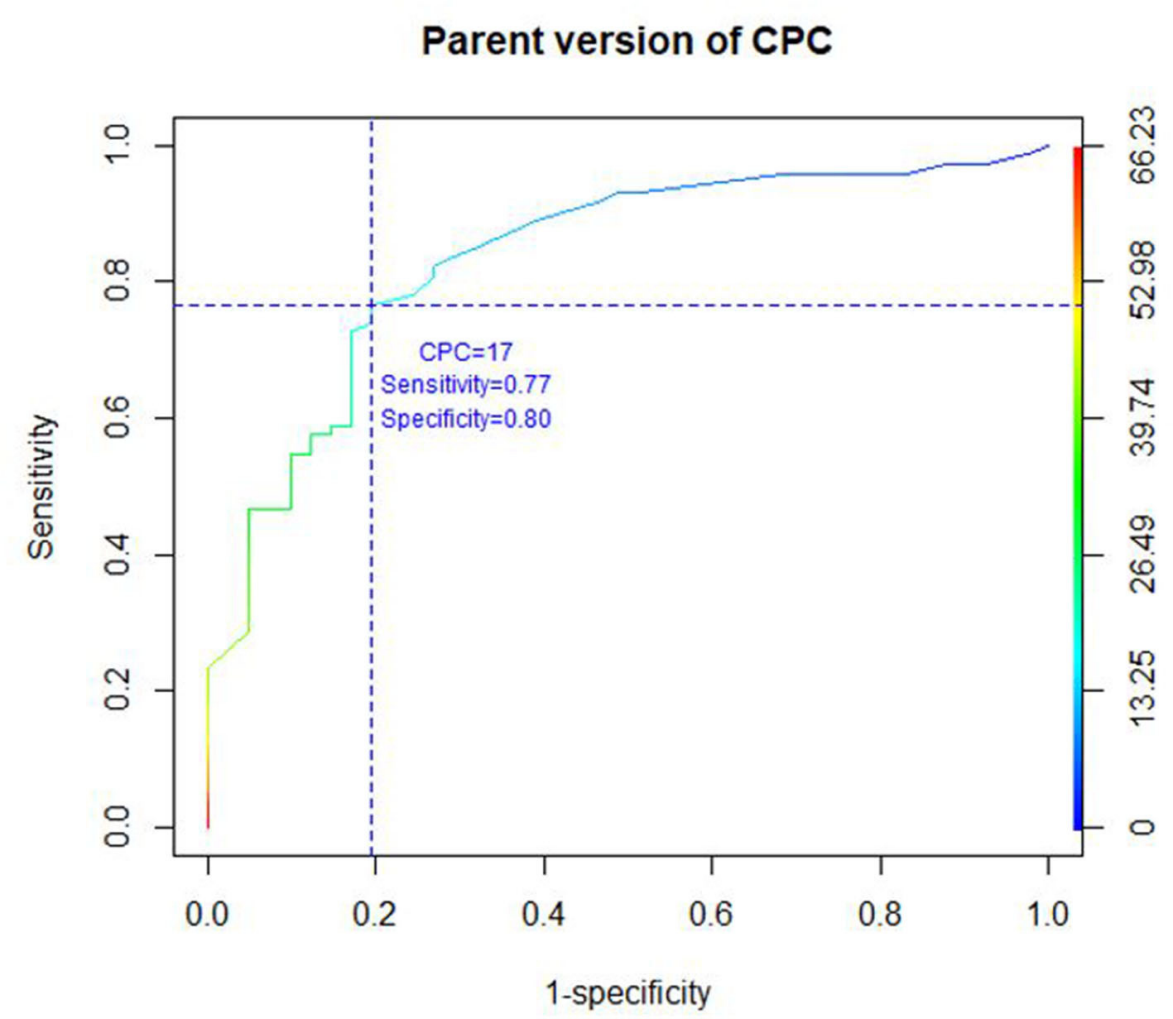
ROC CURVE parent version of CPC.

Taking the K-SADS-PL as the diagnostic reference, with a diagnostic cutoff of ≥20 for child version as recommended by Scheeringa (11), the sensitivity and specificity of the child version at that threshold were 73.1 and 84.7%, respectively (Figures 1, 2). Concerning the parent/caregiver version, using the cutoff of ≥17, the sensitivity and specificity were 76.7 and 80.5%, respectively. The sensitivity and specificity for both versions at various cutoff scores can be calculated from the ROC curve coordinates (figX_1_ & X_2_). The area under the curve was 0.88 for the child version and 0.84 for the parent/caregiver version.

## Discussion

Results suggest that the CPC exhibits good psychometric properties (internal consistence, concurrent validity, and factorial validity) in French-speaking school-aged children.

In this French version, the internal consistency was good (Cronbach's alpha 0.90 for the child version and 0.91 for the parent version). In the Olliac study, Cronbach's alpha was 0.87 for the CPTS-RI, with variations between 0.91 and 0.68 depending on the samples (12). However, the internal consistency of the CPC appears to be as good as that of the CPTS-RI.

For the child version, the two-factor structure of our French version explained 37 and 6% of the variance, respectively. The first factor consists of the four main symptoms of PTSD: items exploring reexperiencing of the event, avoidance, alteration of cognition and mood, and overactivation. It explains 37% of the variance. The second factor included the symptoms of imprudence and represented 6% of the variance. With an observed variance of 43% explained by the four factors, the French CPC seems to be a valid tool (17).

Item 11 (concerning negative beliefs), item 12 (unwanted false thoughts concerning the traumatic event), and item 13 (anhedonia) have been added in the DSM-5. It seems to be central symptoms of the pediatric PTSD.

Negative beliefs and false thoughts, in the DSM-5, refer to a change in the child's belief for himself, the world, or other people (18): “Persistent and exaggerated negative expectations about one's self, others, or the world (e.g., ‘I am bad,' ‘no one can be trusted,' ‘I've lost my soul forever,' ‘my whole nervous system is permanently ruined,' ‘the world is completely dangerous')” (2). Studies have shown that this symptom correlates positively with the presence of PTSD in children (19). In addition, it appears that these negative beliefs refer to maladaptive responses to the psychotrauma experienced and could be involved in the development of internalized symptoms (20).

Anhedonia refers to a loss of interest in previously enjoyed activities and a decrease in the ability to experience pleasure (21). Recent studies suggest that anhedonia is a transdiagnostic construct (22–25). It is also frequently seen in other neuropsychiatric disorders with which depression is commonly comorbid, such as for example obsessive-compulsive disorder (26) or PTSD (27). Anhedonia appears to be more prevalent in girls than in boys (28). Cumulative traumatic experiences increase anhedonia in PTSD (29). There are also strong associations between anhedonia and dissociative symptoms in children (30).

The results obtained for the PCA indicate that CPC explains as well the observed variance of the CPTS-RI (43% with two factors vs. 44.8% with three factors). The main symptoms are found globally in factor 1 and explain a significant percentage of variance for the two scales. On the other hand, the CPC is more refined for the other factors, due to the inclusion of child-specific symptoms (e.g., negative beliefs or false thoughts) that have been added in the DSM-5.

The main limitation of this study concerns the sample. Indeed, this research was offered to children who lived on July 14, 2016, with or without PTSD. As a result, other studies will have to be carried out in order to test the psychometric properties of this scale, in particular on repeated trauma (e.g., maltreatment or witnessing domestic violence). Furthermore, the number of subjects analyzed is less than the number of subjects needed according to Garson (31). Nevertheless, the results seem robust and statistically significant. We also limited the heterogeneity by analyzing the scores of patients with the same traumatic event.

## Conclusion

The investigation of PTSD according to DSM-5 may be challenging in children and adolescents. The French version of the CPC is quickly and easily administrated and scored. Its psychometric properties make it a valuable self-administered tool for clinicians and researchers to assess PTSD symptoms in the pediatric population.

## Data Availability Statement

Publicly available datasets were analyzed in this study. This data can be found at: gindt.m@pediatrie-chulenval-nice.fr.

## Ethics Statement

The studies involving human participants were reviewed and approved by The National Ethics Committee NORD OUEST III (number 2017-A02212-51). Written informed consent to participate in this study was provided by the participants' legal guardian/next of kin.

## Author Contributions

MG, MB, ST, and FA: conceived and designed the experiments. MG, AR, and MB: performed the experiments. MG, AR, and RF: analyzed the data. MG, AR, AF, and FA: wrote the first draft of the manuscript. All authors contributed to manuscript revision, read, and approved the submitted version.

## Conflict of Interest

The authors declare that the research was conducted in the absence of any commercial or financial relationships that could be construed as a potential conflict of interest.

## Publisher's Note

All claims expressed in this article are solely those of the authors and do not necessarily represent those of their affiliated organizations, or those of the publisher, the editors and the reviewers. Any product that may be evaluated in this article, or claim that may be made by its manufacturer, is not guaranteed or endorsed by the publisher.
